# Design and Synthesis of New Quinoxaline Derivatives as Anticancer Agents and Apoptotic Inducers

**DOI:** 10.3390/molecules24061175

**Published:** 2019-03-25

**Authors:** Aliya M. S. El Newahie, Yassin M. Nissan, Nasser S. M. Ismail, Dalal A. Abou El Ella, Sohair M. Khojah, Khaled A.M. Abouzid

**Affiliations:** 1 Pharmaceutical Organic Chemistry Department, Faculty of Pharmacy, October University for Modern Science and Arts (MSA), Cairo 12611, Egypt; anewahie@msa.eun.eg; 2 Pharmaceutical Chemistry Department, Faculty of Pharmacy, Cairo University, Cairo 11562, Egypt; yassin.nissan@hotmail.com; 3 Pharmaceutical Chemistry Department, Faculty of Pharmacy, October University for Modern Science and Arts (MSA), Cairo 12611, Egypt; 4 Pharmaceutical Chemistry Department, Faculty of Pharmaceutical Sciences and Pharmaceutical Industries, Future University in Egypt, Cairo 12311, Egypt; saadnasser2003@yahoo.com; 5 Pharmaceutical Chemistry Department, Faculty of Pharmacy Ain Shams University, Abbassia, Cairo 11566, Egypt; dalal999@hotmail.com; 6 Department of Pharmaceutical Chemistry, Faculty of Pharmacy, Nahda University, Beni Suef 62513, Egypt; 7 Biochemistry Department, Faculty of Science, King Abdulaziz University, Jeddah 21589, Kingdom of Saudi Arabia; smkhojah@yahoo.com; 8 Department of Organic and Medicinal Chemistry, Faculty of Pharmacy, University of Sadat City, Menoufia 32897, Egypt

**Keywords:** quinoxaline, synthesis, anti-cancer activity, cell cycle

## Abstract

The quinoxaline scaffold is a promising platform for the discovery of active chemotherapeutic agents. Three series of quinoxaline derivatives were synthesized and biologically evaluated against three tumor cell lines (HCT116 human colon carcinoma, HepG2, liver hepatocellular carcinoma and MCF-7, human breast adenocarcinoma cell line), in addition to VEGFR-2 enzyme inhibition activity. Compounds **VIId**, **VIIIa**, **VIIIc**, **VIIIe** and **XVa** exhibited promising activity against the tested cell lines and weak activity against VEGFR-2. Compound **VIIIc** induced a significant disruption in the cell cycle profile and cell cycle arrest at the G2/M phase boundary. In further assays, the cytotoxic effect of the highly active compounds was determined using a normal Caucasian fibroblast-like fetal lung cell line (WI-38). Compound **VIIIc** could be considered as a lead compound that merits further optimization and development as an anti-cancer and an apoptotic inducing candidate against the HCT116 cell line.

## 1. Introduction

Cancer is a major human health problem that remains the second highest cause of mortality worldwide [1], where 1,688,780 new cancer cases and 600,920 cancer deaths were projected to occur in the United States in 2017 [2]. Human protein tyrosine kinases (PTKs) play a central role in human carcinogenesis [3], whereas cell cycle progression, cell division and proliferation are viewed as a sequence of events controlled by a cascade of those protein kinases, so PTKs have emerged as promising new cancer therapy targets [4]. Quinoxalines are considered as an important basis for anti-cancer drugs as they are proved to be selective adenosine triphosphate (ATP) competitive inhibitors in many kinases [5] for example: vascular endothelial growth factor receptor (VEGFR), platelet-derived growth factor receptor (PDGFR), proto-oncogene non-receptor tyrosine-protein kinase (Src), c-Met proto-oncogene (c-Met kinase), epidermal growth factor receptor\human epidermal growth factor receptor (EGFR/HER-2), Janus kinase receptor (JAK-2), FMs-related tyrosine kinase 3 (FLT-3) and cyclin dependent kinase (CDK1,2,4,6) [6]. In 2016, Zghaib et al. reported that imidazo[1,2-*a*]quinoxaline derivatives were major microtubule-interfering agents with potent anticancer activity [7]. Quinoxalines with amide and sulphonamide moieties have been reported to inhibit the growth of human tumor cell lines [8]. 

**Figure 1:**
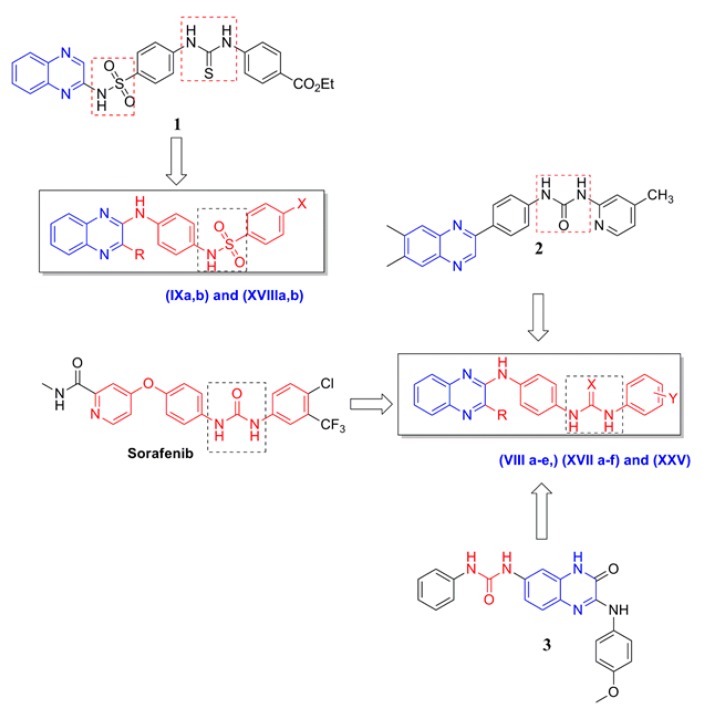
Design of the synthesized compounds **IXa**,**b**), (**XVIIIa**,**b**, **VIII a**–**e**, **XVII a**–**f** and **XXV**.

**Figure 2:**
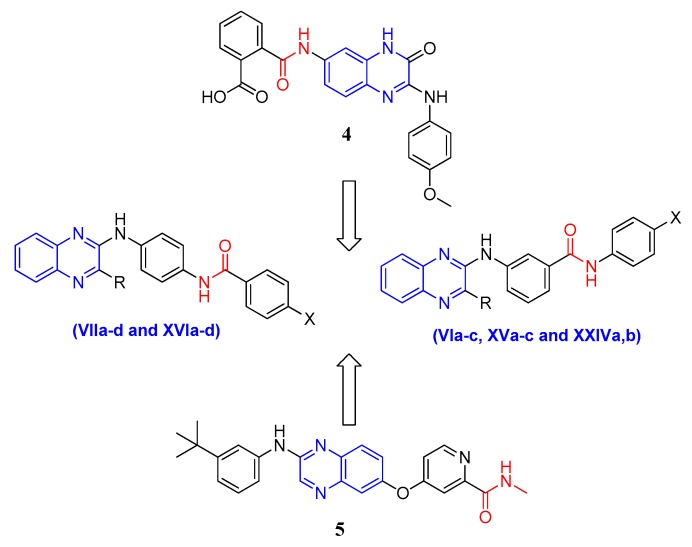
Design of the synthesized compounds **VII a**–**d**, **XVI a**–**d**, **VI a**–**c**, (**XV a**–**c** and **XXIV a**,**b**).

Ghorab et al. [9] designed (quinoxalin-2-yl)benzene sulphonamide derivative **1** (Figure 1) with a potent anti-cancer activity against human liver cancer cell line (Hep G2). Quinoxaline-bisarylurea **2** (Figure 1) has been reported by Göring et al. [10] to exhibit anti-tumor activity. Additionally, quinoxalines were found to induce apoptosis as a mechanism for their anti-cancer activity [11,12]. In 2014, Shahin et al. [13] designed a series of new quinoxaline-based scaffolds bearing amide, sulphonamide and urea moieties which were biologically evaluated for their inhibitory activity against VEGFR-2. In that study, compound **3** (Figure 1) displayed the best IC_50_ value of 10.27 µM, while compound **4** (Figure 2) displayed the best inhibition percentage against VEGFR-2 which was 69% [6]. Furthermore, quinoxaline **5** having an amide group (Figure 2) has been also reported by Ramurthy et al. as a potent Rapidly Accelerated Fibrosarcoma (Raf) kinase inhibitor [14]. Finally, quinoxalines were recently reported to exhibit inhibition against MCF-7 (83.3%) and HCT116 (70%) cell lines [15].

The antitumor activity of kinase inhibitors containing a diaryl urea scaffold has been gaining great attention as they possess a unique binding mode and obvious kinase inhibition profile [16]. Sorafenib is a well-known diarylurea multi-targeted inhibitor of several kinases including Raf, VEGFR, c-Kit and PDGFR [17].

Based on the abovementioned anti-proliferative quinoxaline compounds **1**–**5** and sorafenib as a urea derivative [18], we aimed to design a new series of quinoxaline-based compounds with amide, urea, thiourea and sulphonamide moieties. Hence, linking pharmacophoric functionalities to different aryl moieties promises to have anti-proliferative and apoptotic inducing activity. The design of the target compounds is presented in Figure 1 and Figure 2.

## 2. Results and Discussion

### 2.1. Chemistry

**Scheme 1:**
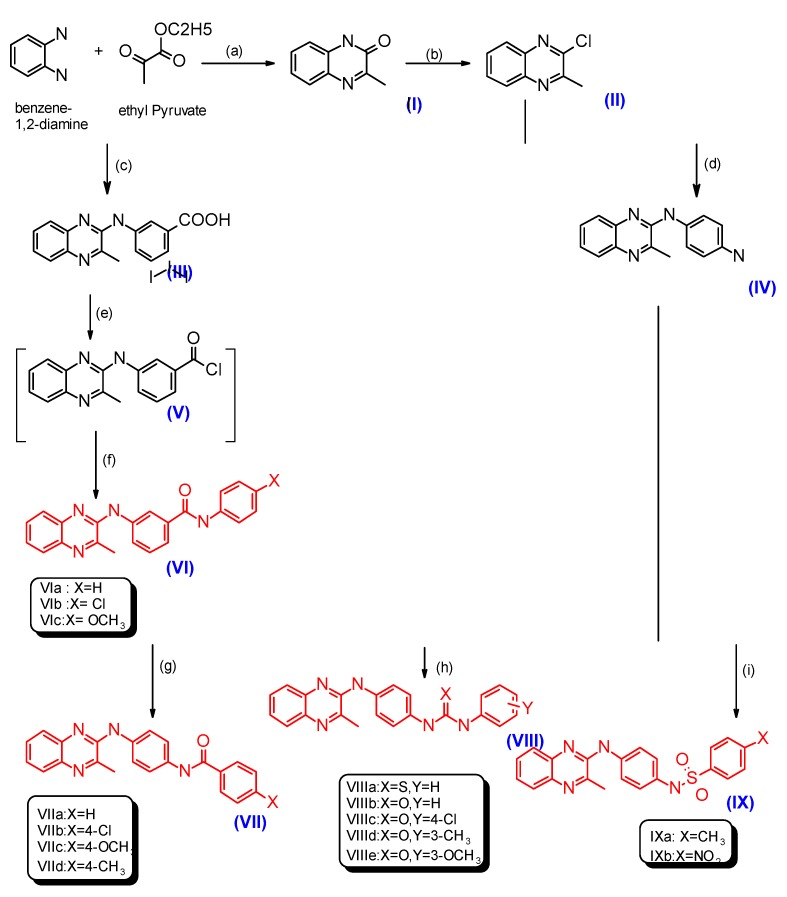
Synthetic pathways for compounds **VI**–**IX**. Conditions and Reagents: (a): *n*-BuOH reflux For 1 h, 97%, (b): POCl_3_ 1.5 h, 70%, (c): *m*-amino benzoic acid in BuOH, conc HCl reflux 4 h, 75%, (d): *p*-phenylene diamine reflux at 110 °C, 5 h, 70%, (e): SOCl_2_ in dry benzene reflux 5 h, 60%, (f): aromatic amines, TEA in dry DCM, reflux 4 h, (g): aromatic acid chlorides in dry toluene reflux 2 h, (h): phenyl isocyanates/isothiocyanates in dry toluene, reflux 5 h, (i): benzene sulphonyl chlorides in dry pyridine reflux 2 h.

**Scheme 2:**
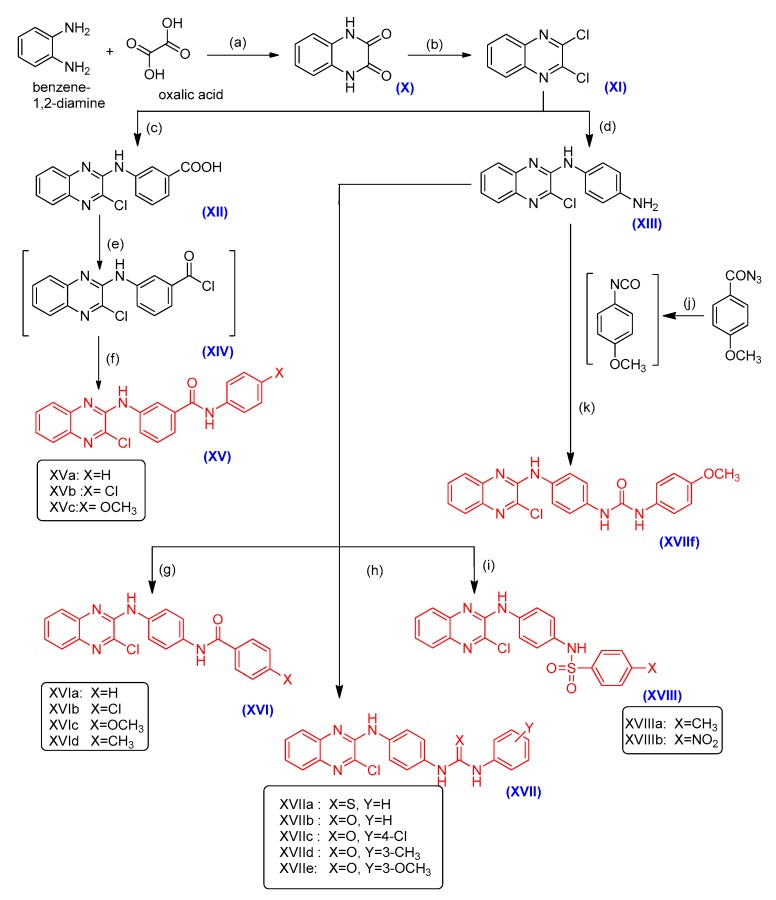
Synthetic pathways for compounds **XV**, **XVIIf**, **XVI–XVIII**. Reagents and Conditions: (a): Aq. HCl, reflux 2 h, 97.3%, (b): POCl_3,_ reflux 12 h, 73.7%, (c): *m*-aminobenzoic acid in *n*-BuOH, conc HCl, reflux 5 h, 75%, (d): *p*-phenylenediamine, reflux at 110 °C, 5 h, 65%, (e): SOCl_2_ in dry benzene, reflux 5 h, 65%, (f): aromatic amines, TEA in dry DCM, reflux 4 h, (g): aromatic acid chlorides in dry toluene, reflux 2 h, (h): phenyl isocyanates/isothiocyanates in dry toluene, reflux 5 h, (i): benzenesulphonyl chlorides in dry pyridine, reflux 2 h, (j): Toluene, 60° C 30 min, (k): Ar-NH_2_, dry toluene, reflux 5 h.

**Scheme 3:**
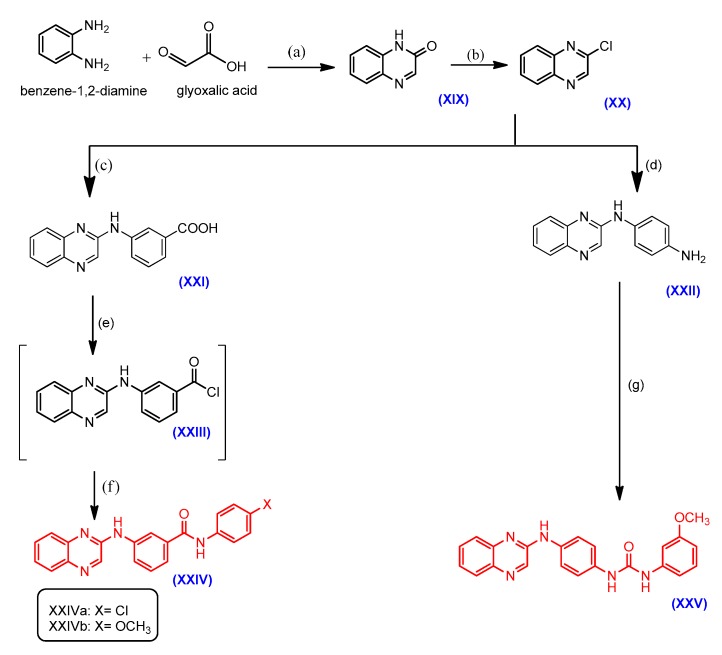
Synthetic pathways for compounds **XXIV** and **XXV**. Conditions and Reagents: (a): Glyoxalic acid in *n*-BuOH reflux for 5 h, 76%, (b): POCl_3_, PCl_5_, reflux 4 h, 46%, (c): *m*-amino- benzoic acid in *n*-BuOH, conc. HCl, reflux 5 h, 75%, (d): *p*-phenylenediamine, reflux at 110 °C, 5 h, 65%, (e): SOCl_2_ in dry benzene, reflux, 5 h, 64%, (f): aromatic amines, TEA in dry DCM, reflux, 4 h, (g) *m*-methoxyphenyl isocyanate in dry toluene, reflux, 5 h, 61%.

Quinoxalines are prepared by the reaction of *o*-phenylenediamine and α-ketocarboxylic acids [19] through different methods. The synthesis of the starting compounds **I**, **X** and **XIX** is depicted in Scheme 1, Scheme 2 and Scheme 3, respectively.

3-Methylquinoxalin-2(1*H*)-one (**I**) and 2(1*H*)-quinoxalinone (**XIX**) could be obtained by the reaction of *o*-phenylenediamine with ethyl pyruvate (Westphal et al. [20]) for **I** or with glyoxylic acid (for **XIX**) in *n*-butanol [21,22]. The treatment of substituted *o*-phenylenediamines with oxalic acid in 4N hydrochloric acid under reflux quickly afforded quinoxaline-2,3(1*H*,4*H*)-dione (**X**) [23,24]. The starting compounds were then subjected to chlorination by treatment with POCl_3_ [20,21,25] alone (compounds **I**, **X**) or with PCl_5_ (compound **XIX**) [26] affording compounds **II, XI** and **XX** in a pure form with melting points as reported.

The chloroquinoxalines were further refluxed with *m*-aminobenzoic acid in *n*-butanol [27], the solution was left to cool and excess *m*-aminobenzoic acid was removed by dissolving in 5% NaOH, then intermediate compounds **III**, **XII** and **XXI** were reprecipitated by dropwise addition of concentrated hydrochloric acid. In our present study, we planned to synthesize target amide compounds **VIa**–**c**, **XVa**–**c** and **XXIVa**,**b**. For the preparation of such compounds, the reaction of the three intermediates **III**, **XII** and **XXI** with the corresponding aromatic amine seemed to be most convenient approach.

In order to achieve such a reaction, the three intermediates were firstly activated as acid chloride derivatives by refluxing with excess thionyl chloride (Wang et al. [28]), then reacting the freshly prepared acid chlorides with the appropriate aromatic amines (aniline, *p*-chloroaniline, *p*-methoxy aniline) in dichloromethane yielding the final products **VIa–c**, **XVa–c** and **XXIVa**,**b** in moderate yields (Kakuta et al. [29]).

In another route, the respective chloroquinoxalines **II**, **XI** and **XX** were refluxed with *p*-phenylenediamine in n-butanol at 110 °C [30] to give high yields of another three important intermediate compounds **IV**, **XIII** and **XXII**, which were further utilized to obtain different target amides **VIIa**–**d**, **XVIa**–**d**, thioureas **VIIIa**, **XVIIa**, ureas **VIIIb**–**e**, **XVIIb**–**e** and **XXV** and sulphonamides **IXa**–**b**, **XVIIIa**–**b**.

The amide derivatives were synthesized from the reaction of intermediate compounds **IV**, **XIII** and **XXII** with different aromatic acid chlorides (*viz;* benzoyl chloride, *p*-chlorobenzoyl chloride, *p*-methoxybenzoyl chloride, *p*-tolylbenzoyl chloride) in diethyl ether (compounds **VIIa**, **XVIa**) or toluene (compounds **VIIb**–**d**, **XVIb**–**d** (Kakuta et al. [29]). The thiourea and urea derivatives **VIIIa**–**e**, **XVIIa**–**e** and **XXV** were obtained by the reaction of intermediate compounds **IV**, **XIII** and **XXII** with phenyl isothiocyanate, phenyl isocyanate, *p*-chloro, *m*-methyl and *m*-methoxyphenyl isocyanates in dry toluene [31]. As for the urea compound **XVIIf**, 4-methoxybenzoyl azide was used as the starting material for the synthesis of *p*-methoxyphenyl isocyanate (via a Curtius rearrangement [32]), which was further reacted with **XIII** in dry toluene to yield the desired compound. Finally, the synthesis of the desired sulphonamides derivatives **IXa**,**b**, **XVIIIa**,**b** was carried out by the reaction of the key intermediate compounds **IV**, **XIII** and **XXII** with a number of arylsulphonyl chlorides (*p*-methylbenzenesulfonyl chloride, *p*-nitrobenzenesulfonyl chloride) in dry pyridine as a solvent [33].

The structures of the newly synthesized compounds were confirmed by elemental analyses and spectral data. ^1^H-NMR spectra for the synthesized amides **VIa**–**c**, **XVa**–**c** and **XXIVa**,**b** showed new signals that appeared downfield in the range δ (11.93–10.58), (11.90–10.47) and (10.53–10.50) ppm assignable to the protons of the newly formed amide, respectively, while the carboxylic acid protons signals disappeared and new aromatic protons appeared in the aromatic range. The spectrum of **XVa** showed one doublet at δ 7.80 ppm and two additional triplet signals at δ 7.40, 7.15 ppm. The spectrum of compound **XVb** displayed two doublets signals at δ 7.84, 7.45 ppm. Compound **XVc** showed two doublet signals at δ 7.70, 6.96 ppm. The data of compound **XXIVa** exhibited two doublet signals at δ 7.07, 6.65 ppm. In addition, an extra singlet signal attributed to the methoxy protons appeared for compounds **VIc**, **XVc** and **XXIVb** at δ 3.77, 3.70 and 3.78 ppm respectively. The ^13^C-NMR spectrum of compound **XVa** showed one signal at δ 164.92 related to the amide group. The FT-IR spectra displayed the amidic carbonyl group (C=O) at 1651–1681 cm^−1^ in lower frequency confirming the conversion of the acidic carboxylic acid (COOH) carbonyls into amides. Compound **XXIVb** revealed a new band corresponding to aliphatic CH groups at 2920 cm^−1^.

On the other hand, the ^1^H-NMR spectra of the corresponding amides **VIIa**–**d** and **XVIa**–**d** showed an increase in the integration of aromatic protons corresponding to the additional unsubstituted and substituted phenyl rings. The spectrum of compound **VIIb** showed additional deshielded aromatic protons as two doublets at δ 8.02 and 7.95 ppm. Compounds **VIIc** and **XVIc** spectrum showed additional deshielded aromatic protons as two doublets at δ (8.00, 7.08 ppm) and (8.20, 7.09 ppm) respectively, in addition to a new single signal at δ 3.80 and 3.88 ppm corresponding to methoxy protons. The spectra of compounds **VIId** and **XVId** showed an additional singlet signal at δ 2.42 and 2.40 ppm, corresponding to the three protons of the newly formed methyl group. Also, the ^13^C-NMR spectruma of **VIId** showed two signals at δ 165.00 and 21.49 ppm related to the newly formed amide and methyl groups, respectively. The FT-IR spectra displayed the amidic carbonyl group (C=O) at 1636–1650 cm^−1.^

Concerning the urea and thiourea final compounds **VIIIa**–**e**, **XVIIa**–**f** and **XXV**, the corresponding ^1^H-NMR spectra showed two extra signals of equal integration that appeared downfield corresponding to two D_2_O exchangeable protons of the urea group in the range of δ (10.36–8.57), (11.00–8.63) and (9.67–9.57) ppm, respectively. An extra singlet signal at δ 2.27, 2.29 ppm appeared corresponding to the methyl protons for **VIIId** and **XVIId**, in addition to a singlet signal at δ 3.74, 3.68 and 3.86 ppm attributed to the methoxy protons for **VIIIe**, **XVIIe** and **XVIIf** respectively. Elucidation of the structures of the target sulfonamide derivatives **IXa**,**b** and **XVIIIa**,**b** was also proved by the increase in the integration of aromatic protons. The spectra of compounds **IXa** and **XVIIIa** showed new singlet signals equivalent to the methyl protons that appeared at δ 2.32 and 2.37 ppm, respectively. The spectrum of compound **IXb** showed additional aromatic protons as two doublets at δ 8.22 and 7.95 ppm. Interestingly, the ^13^C-NMR spectra of **VIIIc**, **VIIIe** showed two signals at δ 154.00 and 160.00 ppm, respectively, corresponding to the newly formed urea, in addition to a signal at δ 55.00 ppm related to the methoxy group of **VIIIe**. The IR spectra of the sulphonamide compounds showed the two characteristic SO_2_ bands at around 1332–1347 and 1159–1165 cm^−1^.

### 2.2. Biological Evaluation

#### 2.2.1. In Vitro Cell Proliferation Assay

The targeted compounds were tested for their in-vitro anti-cancer activity against three tumor cell lines (HCT116, Hep G2 and MCF-7). Doxorubicin was included in the experiments as a reference cytotoxic compound. Exponentially growing cells from different cancer cell lines were trypsinized, counted and seeded at the appropriate densities into 96-well microtiter plates. Cells then were incubated in a humidified atmosphere at 37 °C for 24 h. Then, cells were exposed to different concentrations of compounds for 72 h. Then the viability of treated cells was determined using MTT technique (described latter).

As shown in Table 1, Table 2, Table 3 and Table 4, the different series of synthesized compounds displayed different anticancer activity with the IC_50_ values as low as 2.5 µM. The anti-proliferative activity for compounds **VIa**–**c**, **XVa**–**c** and **XXIVa**,**b** bearing a *N*-(phenyl)-3-(quinoxalin-2-ylamino) benzamide moiety towards the three cancer cell lines varies significantly. Compound **XVa** exhibited good anti-cancer activity against tested cell lines but its activity against HCT116 and MCF-7 was more prominent (IC_50_ = 4.4, 5.3 µM, respectively). Compound **XVb** exhibited moderate to weak activities against the tested cell lines, whereas, the rest of the compounds (**VIa**, **VIb**, **VIc**, **XVc**, **XXIVa** and **XXIVb**) showed no significant inhibitory activity.

SAR studies for this series of amide derivatives revealed that having a quinoxaline (compounds **XXIVa,b**) or methyl quinoxaline (compounds **VIa**–**c**) as ring A as diminished the activity towards the three cell lines. The introduction of a small electron withdrawing group such as a chloro (compounds **XVa,b**) into ring A slightly improved the activity. The highest activity was observed, within this series, when ring B was a phenyl group (compound **XVa**). The presence of an electron-withdrawing group in ring B (**XVb**) slightly decreased the activity, while an electron donating group (compound **XVc**) resulted in an inactive compound, as shown in Table 1.

As for compounds **VIIa**–**d** and **XVIa**–**d** bearing a *N*-(4-(quinoxalin-2-yl)amino)phenyl) benzamide moiety, the following results were observed: compound **VIId** exhibited its optimum inhibitory activity against the HCT-116 cell line (IC_50_ = 7.8 µM), while it showed moderate activity against the Hep G2 cell line and the least activity was observed against the MCF-7 cell line. Compounds **VIIa** and **VIIc** showed moderate to weak activity against the tested cell lines. Compound **VIIb** showed weak activity towards the HCT116 cell line and no inhibitory activity towards the Hep G2 and MCF-7 cell lines. Finally, compounds **XVIa**–**d** showed no inhibitory activity.

On the contrary to the previously mentioned SAR studies, the second series of amides **XVIa**–**c** having an electron withdrawing group (chloro) as R showed no significant activity towards the three cell lines, while an electron donating group such as methyl (compounds **VIIa**–**d**) exhibited moderate activity. It was found that among the methyl quinoxaline derivatives the activity increased when ring B was substituted by an electron donating group (compounds **VIIc**,**d**) and decreased when substituted by an electron withdrawing group (compound **VIIb**) as shown in Table 2.

Concerning compounds **VIIIa**–**e**, **XVIIa**–**f** and **XXV** bearing a 1-(phenyl)-3-(4-(quinoxalin-2-ylamino)phenyl)urea and thiourea moieties. Compound **VIIIc** showed the best anti-cancer activity against the HCT116 and MCF-7 cell lines (IC_50_ = 2.5, 9 µM, respectively), but it exhibited moderate activity towards the Hep G2 cell line. Compound **VIIIa** exhibited good anti-cancer activity against the Hep G2 cell line (IC_50_ = 9.8 µM), while it showed moderate activity towards the HCT116 and MCF-7 cell lines. Compound **VIIIe** showed good anti-cancer activity against the HCT116 cell line (IC_50_ = 8.4 µM), but moderate activity towards the Hep G2 and MCF-7cell lines. Compound **VIIId** possessed moderate anti-cancer activity against the HCT-116 cell line but weak activity towards the Hep G2 and MCF-7 cell lines. Compound **VIIIb** showed moderate activity against the MCF-7 cell line, weak activity against the HCT116 cell line and no significant inhibitory activity towards the Hep G2 cell line. Compounds **XVIIe** and **XXV** showed weak activity towards the tested cell lines. Finally, compounds **XVIIa**–**d** and **XVIIf** showed no inhibitory activity against the tested cell lines.

Concerning SAR studies for urea and thiourea derivatives, when ring A was a quinoxaline or chloroquinoxaline the activity ranged from weak to no activity towards the three cell lines, whereas, the methylquinoxaline derivatives were found to be more active. A methylquinoxaline with a thiourea derivative and a benzene ring as ring B (compound **VIIIa**) was the most potent towards Hep G2 and it lost its activity by 10-fold when converted into the corresponding urea derivative **VIIIb**. However, replacement of the benzene ring in ring B of that urea derivative with a *p*-chlorobenzene (compound **VIIIc**) resulted in an improvement of its activity. Interestingly, adding a *meta* electron- donating group (compounds **VIIId**, **VIIIe**) instead of a *para* electron withdrawing group (compound **VIIIc**) slightly decreased the activity as shown in Table 3.

Finally, within compounds bearing a *N*-(4-(quinoxalin-2-yl)amino)phenyl)-substituted benzene sulfonamide moiety (compounds **IXa**,**b** and **XVIIIa**,**b**), the methylquinoxaline benzene sulfonamide derivatives **IXa**,**b** were more potent than that of chloroquinoxalines (compounds **XVIIIa**,**b**). Substitution of ring B with a *para* electron withdrawing group (compound **IXb**) was more favorable than substitution with a *para* electron donating group (compound **IXa**) as shown in Table 4.

The overall results from the cell lines’ point of view revealed that the three 3-(methylquinoxalin-2-yl)amino derivatives **VIId**, **VIIIc** and **VIIIe** were active as anti-cancer agents against the HCT116 cell line (IC_50_ = 7.8, 2.5 and 8.4 µM) respectively, while only one compound belonging to the 3-(chloroquinoxalin-2-yl)amino group (compound **XVa**) showed marked anti-cancer activity against the HCT116 cell line (IC_50_ = 4.4 µM). Derivatives belonging to the unsubstituted quinioxalin-2-yl-amino group didn’t exhibit any anti-cancer activity. Regarding the Hep G2 and MCF-7 cell lines, the majority of the screened compounds did not show any remarkable anti-cancer activity against both cell lines except compound **VIIIa** against the Hep G2 cell line (IC_50_ = 9.8 µM) and both compounds **VIIIc** and **XVa** against the MCF-7 cell line (IC_50_ = 9.0, 5.3 µM), respectively.

#### 2.2.2. In Vitro VEGFR-2 Inhibition Assay

In an attempt to investigate the possible mechanism of action of the synthesized compounds based on structural similarity with sorafenib, all of them were tested for VEGFR-2 inhibitory activity at Bio Science Corporation (BPS, San Diego, CA, USA). The assay was performed using the Kinase-Glo Plus luminescence kinase assay kit (Promega, San Diego, CA, USA). It measures kinase activity by quantitating the amount of ATP remaining in solution following a kinase reaction. The percent inhibition of the enzymatic activities caused by our compounds against VEGFR-2 was evaluated against a reference kinase inhibitor (staurosporine) at a single concentration of 10 µM. Unfortunately, our compounds showed weak activity against VEGFR-2, where most of the compound’s activity was between 2% and 21% as illustrated in Table 5.

#### 2.2.3. Cell Cycle Analysis

**Figure 3:**
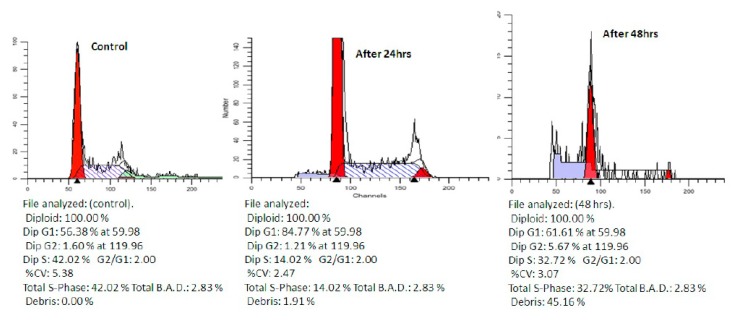
The effect of compound **VIIIc** on the normal cell cycle progression in HCT116 cells.

**Figure 4:**
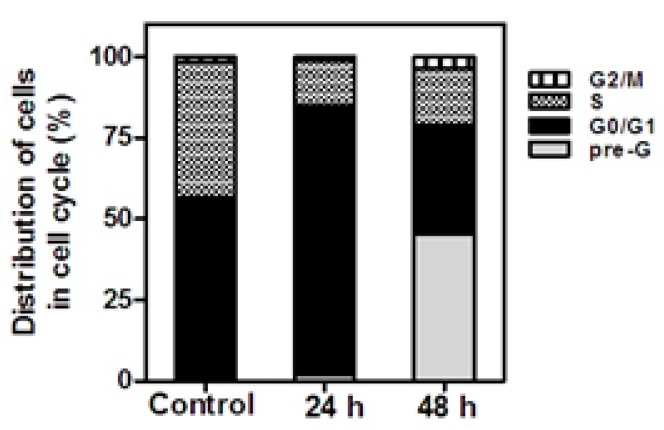
Exposure of HCT116 cells to **VIIIc** at GI_50_ concentration (0.36 µM) for 24 h and 48 h.

In another attempt to study the effect of the synthesized quinoxalines that exhibited potent antiproliferative activity against the three NCI cell lines (HCT116, Hep G2 and MCF-7), on cell cycle progression and induction of apoptosis, compound **VIIIc** was selected as being the most active compound for this study on the human colon carcinoma cell line HCT116. The effect of compound **VIIIc** on the normal cell cycle progression was characterized using flow cytometric analysis of the DNA ploidy in HCT116 cells Figure 3. Exposure of HCT116 cells to **VIIIc** at IC_50_ concentration (2.5 µM) for 24 h resulted in an increase in G1 phase and after 48 h a significant disruption in cell cycle profile and cell cycle arrest at G2/M phase boundary with concurrent time dependent increase in pre-G cell population Figure 4. The observed increase in pre-G cell population may imply DNA fragmentation and apoptosis as a potential mechanism for **VIIIc**-induced cancer cell death.

#### 2.2.4. Apoptosis Determination

**Figure 5:**
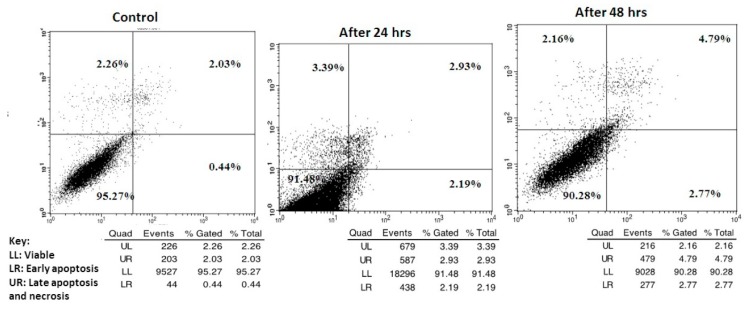
Exposure of HCT116 cells to **VIIIc** at its IC_50_ (0.36 µM) for 24 h and 48 h increased the percentage of annexin-V positive cells indicating an early (lower right quadrant) or late (upper right quadrant) apoptosis in a time dependent manner compared to dimethylsulphoxide (DMSO) treated cells.

For further investigation of the ability of compound **VIIIc** to induce apoptosis, an Annexin V (conjugated to FITC) apoptosis detection kit was employed. This assay detects phosphatidylserine (PS) expressed on the surface of apoptotic cells and fluoresces green after interacting with the labeled annexin V. During early apoptosis, membrane asymmetry is lost, and PS translocates from the cytoplasmic side of the membrane to the external leaflet. Propidium iodide (PI), the counterstain used in this assay, has the ability to cross only compromised membranes to intercalate into the DNA. Therefore, PI is used to detect the late stages of apoptosis by presence of red fluorescence. Exposure of HCT116 cells to **VIIIc** at its IC_50_ (2.5 µM) for 24 h and 48 h increased the percentage of annexin-V positive cells indicating an early (lower right quadrant) or late (upper right quadrant) apoptosis in a time dependent manner compared to dimethylsulphoxide (DMSO) treated cells Figure 5.

#### 2.2.5. In Vitro Cytotoxic Assay

In an attempt to investigate the cytotoxic potential of our designed compounds, MTT cytotoxicity assay was performed for the most active compounds **VIId**, **VIIIa**, **VIIIc**, **VIIIe** and **XVa** on Caucasian fibroblast-like fetal lung cell line (WI-38) using staurosporine as a reference compound. The results revealed that compounds **XVa**, **VIIIa** were highly selective anti-cancer agents having IC_50_ of (163.6, 126.19 µM) respectively. Compounds **VIIIc** and **VIIIe** showed moderate selectivity having IC_50_ of (97.2, 96.4 µM) respectively. Lastly, compound **VIId** exhibited low selectivity having IC_50_ of (40.13 µM) as shown in Table 6.

## 3. Materials and Methods

### 3.1. General Information

The reactions were monitored and the purities of the compounds were checked by ascending thin layer chromatography (TLC) on silica gel-coated aluminum plates (60 F_254_, 0.25 mm, Merck, Darmstadt, Germany) using mixture of chloroform and methanol (1:9) and the spots were visualized under ultra violet light at 254 and 366 nm. Melting points were determined in open capillaries using a Stuart (Biocote, Staffordshire, ST15 OSA, UK) scientific melting point apparatus without correction. EI-MS spectra were recorded on a Finnigan Mat SSQ 7000 (70 ev) mass spectrometer (Ringoes, NJ, USA) at Regional Center for Mycology and Biotechnology at El Azhar University. ^1^H-NMR spectra were recorded on the δ scale given in ppm on a Bruker 400 MHz spectrophotometer (Billerica, MA, USA) referred to TMS using DMSO-*d*_6_ as solvent. Chemical shifts (δ) were expressed in parts per million (ppm) and coupling constants (*J*) were expressed in Hertz (Hz). The signals were designated as follows: s, singlet; d, doublet; t, triplet; m, multiplet at Microanalytical Center unit FOPCU at Cairo University. FT-IR spectra were determined (KBr) using a IR-435 (Shimadzu, Kyoto, Japan) or FT-IR 1650 (Perkin Elmer, Waltham, MA, USA) spectrometer at the Faculty of Pharmacy, Cairo University. Elemental analyses were performed at Regional Center for Mycology and Biotechnology at El-Azhar University.

The synthesis of the starting compounds **I**, **X** and **XIX** according to the previously reported method [20,21,22,23,24].

### 3.2. Chemistry 

#### 3.2.1. General Procedures for the Synthesis of Compounds **VIa**–**c**, **XVa**–**c** and **XXIVa,b**

A mixture of intermediates **III**, **XII** and **XXI** (1.8 mmol) and thionyl chloride (SOCl_2_, 15 mL) was stirred under reflux for 5 h. The mixture was then evaporated under reduced pressure, yielding acid chlorides **V**, **XIV** and **XXIII** [28]. The title amides were afforded by drop wise addition of a solution of the corresponding acid chloride (1.5 mmol) in methylene chloride (5 mL) to a hot solution of excess aromatic amines (aniline, *p*-chloroaniline, *p*-methoxyaniline) in DCM (5 mL). The mixture was left to reflux for 4–6 h till precipitation occurs. The precipitate was filtered while hot, washed with DCM and crystallized from EtOH to afford the title compounds [29].

#### 3.2.2. 3-((3-Methylquinoxalin-2-yl)amino)-*N*-phenylbenzamide (**VIa**)

Brown solid (yield = 65%); m.p. 250–252 °C; ^1^H-NMR (DMSO-*d*_6_) δ: 11.93 (s, 1H, NH amide), 10.53 (s, 1H, NH), 8.39–7.19 (m, 13H, Ar-H), 2.50 (s, 3H, CH_3_). FT-IR (ύ max, cm^−1^): 3356 (2 NH), 2993 (CH aliphatic), 1666 (C=O amide). MS (MWt: 354.4): *m*/*z*, 354.4 [M^+^, (8.16%)], 45.68 (100%); Anal. Calcd for C_22_H_18_N_4_O: C, 74.56; H, 5.12; N, 15.81. Found: C, 71.82; H, 5.18; N, 15.97.

#### 3.2.3. *N*-(4-Chlorophenyl)-3-((3-methylquinoxalin-2-yl)amino)benzamide (**VIb**)

Dark green (yield = 60%); m.p. 240–241 °C; ^1^H-NMR (DMSO-*d*_6_) δ: 11.19 (s, 1H, NH amide), 10.47 (s, 1H, NH), 8.54–7.41 (m, 12H, Ar-H), 2.59 (s, 3H, CH_3_). FT-IR (ύ max, cm^−1^): 3421 (2 NH), 2923 (CH aliphatic), 1652 (C=O amide). MS (MWt: 388.85): *m*/*z*, 390 [M^+^+2, (4.45%)], 388 [M^+^, (0.97%)], 264.03, (100%)]. Anal. Calcd for C_22_H_17_ClN_4_O: C, 67.95; H, 4.41; N, 14.41. Found: C, 68.09; H, 4.45; N, 14.63.

#### 3.2.4. *N*-(4-Methoxyphenyl)-3-((3-methylquinoxalin-2-yl)amino)benzamide (**VIc**) 

Brown solid (yield = 69%); m.p. 255–256 °C; ^1^H-NMR (DMSO-*d*_6_) δ: 10.58 (s, 1H, NH amide), 9.96 (s, 1H, NH), 8.38 (s, 1H, Ar-H), 8.31-6.82 (m, 11H, Ar-H), 3.77 (s, 3H, OCH_3_), 2.51 (s, 3H, CH_3_). FT-IR (ύ max, cm^−1^): 3394 (2 NH), 3055 (CH aromatic), 2997 (CH aliphatic), 1651 (C=O amide). MS (MWt: 384.43): *m*/*z*, 384.07 [M^+^, (5.51%)], 320.13, (100%)]; Anal calcd for C_23_H_20_N_4_O_2_: C, 71.86; H, 5.24; N, 14.57. Found: C, 72.04; H, 5.32; N, 14.72.

#### 3.2.5. 3-((3-Chloroquinoxalin-2-yl)amino)-*N*-phenylbenzamide (**XVa**) 

Creamy white solid (yield = 71%); m.p. > 300 °C; ^1^H-NMR (DMSO-*d*_6_) δ: 10.47 (s, 1H, NH amide), 9.43 (s, 1H, NH), 8.42 (m, 1H, Ar-H), 8.32 (s, 1H, Ar-H), 8.18 (d, *J* = 8 Hz, 1H, Ar-H), 8.02 (d, *J* = 8 Hz, 1H, Ar-H), 7.87 (t, 1H, Ar-H), 7.80 (d, *J* = 8 Hz, 2H, Ar-H), 7.70 (d, *J* = 8 Hz, 1H, Ar-H), 7.62–7.55 (m, 1H, Ar-H), 7.40 (t, 1H, Ar-H), 7.34 (m, 2H, Ar-H), 7.15 (t, 1H, Ar-H); ^13^C-NMR (DMSO-*d*_6_) δ: 164.92, 142.04, 139.37, 137.40, 134.05, 131.02, 130.76, 129.16, 128.58, 127.69, 127.45, 127.12, 124.45, 121.01, 120.91; FT-IR (ύ max, cm^−1^): 3433,3414 (2 NH), 1678 (C=O amide); MS (MWt: 374.82): *m*/*z*, 376.47 [M^+^ + 2, (1.64%)], 374.99 [M^+^, (2.28%)], 113. (100%); Anal. Calcd for C_21_H_15_ClN_4_O: C, 67.29; H, 4.03; N, 14.95. Found: C, 67.48; H, 4.02; N, 15.13.

#### 3.2.6. *N*-(4-Chlorophenyl)-3-((3-chloroquinoxalin-2-yl)amino)benzamide (**XVb**) 

Creamy white solid (yield = 60%); m.p. 283–285 °C; ^1^H-NMR (DMSO-*d*_6_) δ: 10.60 (s, 1H, NH amide), 10.50 (s, 1H, NH), 8.31 (s, 1H, Ar-H), 8.18 (d, *J* = 8 Hz, 1H, Ar-H), 8.03 (d, *J* = 8 Hz, 1H, Ar-H), 7.87–7.85 (m, 1H, Ar-H), 7.84 (d, *J* = 8 Hz, 2H, Ar-H), 7.80–7.78 (m, 2H, Ar-H), 7.57–7.55 (m, 2H, Ar-H), 7.45 (d, *J* = 8 Hz, 2H, Ar-H); FT-IR (ύ max, cm^−1^): 3336 (2 NH), 3062 (CH aromatic), 1681 (C=O amide); MS (MWt: 409.27): *m*/*z*, 413.85 [M^+^ + 4, (0.53%)], 409.20 [M^+^, (27.06%)], 98.03(100%); Anal. Calcd for C_21_H_14_Cl_2_N_4_O: C, 61.63; H, 3.45; N, 13.69. Found: C, 61.58; H, 3.42; N, 13.87.

#### 3.2.7. 3-((3-Chloroquinoxalin-2-yl)amino)-*N*-(4-methoxyphenyl)benzamide (**XVc**) 

Creamy white (yield = 70%); m.p. 240–241 °C; ^1^H-NMR (DMSO-*d*_6_) δ: 11.90 (s, 1H, NH amide), 10.38 (s, 1H, NH), 8.30 (s, 1H, Ar-H), 8.10 (d, *J* = 8 Hz, 1H, Ar-H), 7.90 (d, *J* = 8 Hz, 1H, Ar-H), 7.83 (d, *J* = 8 Hz, 1H, Ar-H), 7.80 (m, 1H, Ar-H), 7.70 (d, *J* = 8 Hz, 2H, Ar-H), 7.50 (m, 1H, Ar-H), 7.20–7.00 (m, 2H, Ar-H), 6.96 (d, *J* = 8 Hz, 2H, Ar-H), 3.70 (s, 3H, OCH_3_); FT-IR (ύ max, cm^−1^): 3356 (2 NH), 3055 (CH aromatic), 2935 (CH aliphatic), 1678 (C=O amide); MS (MWt: 404.85): *m*/*z*, 404.36 [M^+^, (5%)] 44 (100%); Anal. Calcd for C_22_H_17_ClN_4_O_2_: C, 65.27; H, 4.23; N, 13.84. Found: C, 65.54; H, 4.28; N, 14.01.

#### 3.2.8. *N*-(4-chlorophenyl)-3-(quinoxalin-2-ylamino)benzamide (**XXIVa**) 

Buff solid (yield = 75%); m.p. > 300 °C; ^1^H-NMR (DMSO-*d*_6_) δ: 10.53 (s, 1H, NH amide), 10.40 (s, 1H, NH), 8.38 (s, 1H, Ar-H), 8.04 (d, *J* = 8 Hz, 1H, Ar-H), 7.74–7.15 (m, 5H, Ar-H), 7.07 (d, *J* = 8 Hz, 2H, Ar-H), 6.86 (m, 2H, Ar-H), 6.65 (d, *J* = 8 Hz, 2H, Ar-H); FT-IR (ύ max, cm^−1^): 3282 (2 NH), 1681 (C=O amide); MS (MWt:374.8): *m*/*z*, 374.21 [M^+^, (7.82%)], 84.22 (100%); Anal. Calcd for C_21_H_15_ClN_4_O: C, 67.29; H, 4.03; N, 14.95. Found: C, 67.44; H, 4.09; N, 15.08.

#### 3.2.9. *N*-(4-Methoxyphenyl)-3-(quinoxalin-2-ylamino)benzamide (**XXIVb**) 

Dark brown solid (yield = 79%); m.p. > 300 °C; ^1^H-NMR (DMSO-*d*_6_) δ: 10.50 (s, 1H, NH amide), 9.92 (s, 1H, NH), 8.38 (s, 1H, Ar-H), 8.30 (t, 1H, Ar-H), 7.74-7.51 (m, 5H, Ar-H), 7.13–6.72 (m, 4H, Ar-H), 6.65 (d, *J* = 8 Hz, 1H, Ar-H), 6.52 (d, *J* = 8 Hz, 1H, Ar-H), 3.78 (s, 3H, OCH_3_); FT-IR (ύ max, cm^−1^): 3367 (2 NH), 3066 (CH aromatic), 2920 (CH aliphatic), 1651 (C=O amide); MS (MWt: 370.4): *m*/*z*, 371 [M^+^ + H, (1.16%)], 370 [M^+^, (2%)], 119.98 (100%); Anal. Calcd for C_22_H_18_N_4_O_2_: C, 71.34; H, 4.90; N, 15.13. Found: C, 71.48; H, 4.97; N, 15.36.

#### 3.2.10. General Procedures for the Synthesis of Compounds **VIIa**–**d** and **XVIa**–**d**

The title compounds were afforded by dropwise addition of the appropriate benzoyl chlorides (1.2 mmol; 1 eq) (*viz;* benzoyl chloride, *p*-chlorobenzoyl chloride, *p*-methoxybenzoyl chloride, *p*-tolyl-benzoyl chloride) to a hot solution of **IV** or **XIII** (1.2 mmol) in 5 mL of dry diethyl ether (compound **VIIa**, **XVIa**) or 5 mL of dry toluene (compounds **VIIb**–**d**, **XVIb**–**d**). The mixture was left to reflux for 2–4 h till precipitation occured, then the precipitate was filtered while hot, washed with toluene and crystallized from EtOH to afford the title compounds [29].

#### 3.2.11. *N*-(4-((3-Methylquinoxalin-2-yl)amino)phenyl)benzamide (**VIIa**) 

Dark brown solid (yield = 70%); m.p. 210–212 °C; ^1^H-NMR (DMSO-*d*_6_) δ: 10.32 (s, 1H, NH amide), 8.97 (s, 1H, NH), 8.07–7.39 (m, 13H, Ar-H), 2.75 (s, 3H, CH_3_); FT-IR (ύ max, cm^−1^): 3334 (2 NH), 3057 (CH aromatic), 2995 (CH aliphatic), 1645 (C=O amide); MS (MWt.: 354.4): *m*/*z*, 355.12 [M^+^ + H, (13.88%)], 354.12 [M^+^, (57.34%)], 105.05 (100%); Anal. calcd for C_22_H_18_N_4_O: C, 74.56; H, 5.12; N, 15.81. Found: C, 74.58; H, 5.19; N, 16.02.

#### 3.2.12. 4-Chloro-*N*-(4-((3-methylquinoxalin-2-yl)amino)phenyl)benzamide (**VIIb**)

Golden brown solid (yield = 64%); m.p. 234–235 °C; ^1^H-NMR (DMSO-*d*_6_) δ: 10.31 (s, 1H, NH amide), 8.66 (s, 1H, NH), 8.02 (d, *J* = 8 Hz, 2H, Ar-H), 7.95 (d, *J* = 8 Hz, 2H, Ar-H), 7.80 (d, *J* = 8 Hz, 1H, Ar-H), 7.77 (d, *J* = 8 Hz, 2H, Ar-H), 7.68 (d, *J* = 8 Hz, 1H, Ar-H), 7.63 (d, *J* = 8 Hz, 2H, Ar-H), 7.58 (t, 1H, Ar-H), 7.44 (t, 1H, Ar-H), 2.73 (s, 3H, CH_3_); FT-IR (ύ max, cm^−1^): 3441 (2 NH), 2991 (CH aliphatic), 1647 (C=O amide); MS (MWt: 388.8): *m*/*z*, 390.18 [M^+^ + 2, (18.13%)], 388.17 [M^+^, (52%)], 139 (100%); Anal. calcd for C_22_H_17_ClN_4_O: C, 67.95; H, 4.41; N, 14.41. Found: C, 68.12; H, 4.46; N, 14.59.

#### 3.2.13. 4-Methoxy-*N*-(4-((3-methylquinoxalin-2-yl)amino)phenyl)benzamide (**VIIc**) 

Greyish-brown solid (yield = 76%); m.p. 230–231 °C; ^1^H-NMR (DMSO-*d*_6_) δ: 10.58 (s, 1H, NH amide), 8.99 (s, 1H, NH), 8.00 (d, *J* = 8 Hz, 2H, Ar-H), 7.84 (d, *J* = 8 Hz, 2H, Ar-H), 7.80 (d, *J* = 8 Hz, 1H, Ar-H), 7.73 (d, *J* = 8 Hz, 2H, Ar-H), 7.61 (t, 1H, Ar-H), 7.48 (t, 1H, Ar-H), 7.08 (d, *J* = 8 Hz, 2H, Ar-H), 7.00 (d, *J* = 8 Hz, 1H, Ar-H), 3.80 (s, 3H, OCH_3_), 2.76 (s, 3H, CH_3_); FT-IR (ύ max, cm^−1^): 3392 (2 NH), 3032 (CH aromatic), 2935 (CH aliphatic), 1639 (C=O amide); MS (MWt: 384.4): *m*/*z*, 385.19 [M^+^ + H, (3.74%)], 384.18 [M^+^, (14.7%)], 40.16 (100%); Anal. Calcd for C_23_H_20_N_4_O_2_: C, 71.86; H, 5.24; N, 14.57. Found: C, 72.04; H, 5.31; N, 14.71.

#### 3.2.14. 4-Methyl-*N*-(4-((3-methylquinoxalin-2-yl)amino)phenyl)benzamide (**VIId**) 

Black solid (yield = 68%); m.p. 220–222 °C; ^1^H-NMR (DMSO-*d*_6_) δ: 10.15 (s, 1H, NH amide), 9.90 (s, 1H, NH), 8.05 (m, 2H, Ar-H), 7.89 (d, *J* = 8 Hz, 2H, Ar-H), 7.64–7.52 (m, 2H, Ar-H), 7.46–7.43 (m, 1H, Ar-H), 7.41–7.30 (m, 5H, Ar-H), 2.50 (s, 3H, CH_3_), 2.42 (s, 3H, CH_3_); ^13^C-NMR (DMSO-*d*_6_) δ: 165.55, 148.10, 141.94, 136.00, 135.00, 132.00, 129.72, 129.37, 128.15, 127.67, 122.39, 121.17, 22.10, 21.49; FT-IR (ύ max, cm^−1^): 3340 (2 NH), 3032 (CH aromatic), 2916 (CH aliphatic), 1647 (C=O amide); MS: (MWt: 368.43): *m*/*z*, 369.16 [M^+^ + H, (3.17%)], 368.15 [M^+^, (10.27%)], 118.98 (100%); Anal. calcd for C_23_H_20_N_4_O: C, 74.98; H, 5.47; N, 15.21. Found: C, 75.19; H, 5.55; N, 15.34.

#### 3.2.15. *N*-(4-((3-Chloroquinoxalin-2-yl)amino)phenyl)benzamide (**XVIa**) 

Light brown solid (yield = 72%); m.p. 270–273 °C; ^1^H-NMR (DMSO-*d*_6_) δ: 10.29 (s, 1H, NH amide), 9.37 (s, 1H, NH), 8.44–7.24 (m, 13H, Ar-H); FT-IR (ύ max, cm^−1^): 3421 (2 NH), 1636 (C=O amide); MS (MWt: 374.82): *m*/*z*, 374.25 [M^+^, (4.60%)], 57.07 (100%); Anal. Calcd for C_21_H_15_ClN_4_O: C, 67.29; H, 4.03; N, 14.95. Found: C, 67.47; H, 4.11; N, 15.23.

#### 3.2.16. 4-Chloro-*N*-(4-((3-chloroquinoxalin-2-yl)amino)phenyl) benzamide (**XVIb**) 

Orange solid (yield = 66%); m.p. 233–235 °C; ^1^H-NMR (DMSO-*d*_6_) δ: 10.35 (s, 1H, NH amide), 9.61 (s, 1H, NH), 8.04 (d, *J* = 8 Hz, 4H, Ar-H), 7.81 (d, *J* = 8 Hz, 2H, Ar-H), 7.64 (d, *J* = 8 Hz, 2H, Ar-H), 7.60–7.56 (m, 2H, Ar-H), 7.33 (m, 2H, Ar-H); FT-IR (ύ max, cm^−1^): 3320 (2 NH), 1650 (C=O amide); MS (MWt: 409.3): *m*/*z*, 410 [M^+^ + H, (2%)], 409.26[M^+^, (2.8%)], 135 (100%); Anal. Calcd for C_21_H_14_C_l2_N_4_O: C, 61.63; H, 3.45; N, 13.69. Found: C, 61.94; H, 3.5; N,13.78.

#### 3.2.17. *N*-(4-((3-Chloroquinoxalin-2-yl)amino)phenyl)-4-methoxybenzamide (**XVIc**) 

Orange solid (yield = 66%); m.p. 235–236 °C; ^1^H-NMR (DMSO-*d*_6_) δ: 10.15 (s, 1H, NH amide), 9.72 (s, 1H, NH), 8.20 (d, *J* = 7 Hz, 2H, Ar-H), 7.94–7.92 (m, 2H, Ar-H), 7.84 (d, *J* = 8 Hz, 2H, Ar-H), 7.64–7.57 (m, 2H, Ar-H), 7.37–7.31 (m, 2H, Ar-H),7.09 (d, *J* = 8 Hz, 2H, Ar-H), 3.88 (s, 3H, OCH_3_); FT-IR (ύ max, cm^−1^): 3275 (2 NH), 3062 (CH aromatic), 2958 (CH aliphatic), 1643 (C=O amide); MS (MWt: 404.85): *m*/*z*, 406.01 [M^+^ + 2, (2.45%)], 43.96 (100%); Anal. Calcd for C_22_H_17_ClN_4_O_2_: C, 65.27; H, 4.23; N, 13.84. Found: C, 65.42; H, 4.27; N, 13.96.

#### 3.2.18. *N*-(4-((3-Chloroquinoxalin-2-yl)amino)phenyl)-4-methylbenzamide (**XVId**) 

Orange solid (yield = 69%); m.p. 245–246 °C; ^1^H-NMR (DMSO-*d*_6_) δ: 10.21 (s, 1H, NH amide), 9.89 (s, 1H, NH), 8.02–7.83 (m, 6H, Ar-H), 7.64–7.57 (m, 2H, Ar-H), 7.36 (d, *J* = 8 Hz, 4H, Ar-H), 2.40 (s, 3H, CH_3_); FT-IR (ύ max, cm^−1^): 3307,3228 (2NH), 3059 (CH aromatic), 2954 (CH aliphatic), 1637 (C=O amide); MS (MWt: 388.8): *m*/*z*, 89.14 [M^+^ + H, (3.9%)], 388.15 [M^+^, (5.79%)], 277.13 (100%); Anal. Calcd for C_22_H_17_ClN_4_O: C, 67.95; H, 4.41; N, 14.41. Found: C, 68.17; H, 4.49; N, 14.68.

#### 3.2.19. General Procedures for the Synthesis of Compounds **VIIIa**–**e**, **XVIIa**–**f** and **XXV**

The title compounds were afforded by dropwise addition of phenyl isothiocyanate (compounds **VIIIa** and **XVIIa**) or the appropriate phenyl isocyanate (compounds **VIIIb**–**e** and **XVIIb**–**e,** 1.2 mmol; 1 eq) (*viz;* phenyl isocyanate, *p*-chloro-, *m*-methyl- and *m*-methoxyphenyl isocyanate) to a hot solution of **IV**, **XIII** or **XXII** (1.2 mmol) in dry toluene (5 mL). The mixture was refluxed for 3–5 h till precipitation occurs. The precipitate was filtered while hot, washed with toluene and crystallized from EtOH to afford the title compounds [31].

#### 3.2.20. 1-(4-((3-Methylquinoxalin-2-yl)amino)phenyl)-3-phenylthiourea (**VIIIa**) 

Olive green solid (yield = 76%); m.p. 175–176 °C; ^1^H-NMR (DMSO-*d*_6_) δ: 10.36 (s, 1H, NH thiourea), 10.03 (s, 1H, NH thiourea), 8.90 (s, 1H, NH), 8.00–6.82 (m, 13H, Ar-H), 2.70 (s, 3H, CH_3_); FT-IR (ύ max, cm^−1^): 3354 (3 NH), 3053 (CH aromatic), 2950 (CH aliphatic), 1219 (C=S amide); MS (MWt: 385.5): *m*/*z*, 385 [M^+^, (1%)], 93.05 (100%); Anal. calcd for C_22_H_19_N_5_S: C, 68.55; H, 4.97; N, 18.17. Found: C, 68.69; H, 5.03; N, 18.42.

#### 3.2.21. 1-(4-((3-Methylquinoxalin-2-yl)amino)phenyl)-3-phenylurea (**VIIIb**) 

Brown solid (yield = 68%); m.p. > 300 °C; ^1^H-NMR (DMSO-*d*_6_) δ: 9.78 (s, 1H, NH urea), 9.68 (s, 1H, NH urea), 8.65 (s, 1H, NH), 7.95–7.11 (m, 13H, Ar-H), 2.72 (s, 3H, CH_3_); FT-IR (ύ max, cm^−1^): 3356 (3 NH), 3055 (CH aromatic), 2993 (CH aliphatic), 1640 (C=O amide); MS (MWt.: 369.42): *m*/*z*, 370.28 [M^+^ + H, (5.17)],369.29 [M^+^, (7.41%) ], 40.36 (100%); Anal. calcd for C_22_H_19_N_5_O: C, 71.53; H, 5.18; N, 18.96. Found: C, 71.80; H, 5.26; N, 19.18.

#### 3.2.22. 1-(4-Chlorophenyl)-3-(4-((3-methylquinoxalin-2-yl)amino)phenyl)urea (**VIIIc**) 

Greyish-brown solid (yield = 79%); m.p. 248–249 °C; ^1^H-NMR (DMSO-*d*_6_) δ: 8.85 (s, 1H, NH urea), 8.65 (s, 1H, NH urea), 8.57 (s, 1H, NH), 7.86 (d, *J* = 8 Hz, 2H, Ar-H), 7.79 (d, *J* = 8 Hz, 1H, Ar-H), 7.64 (d, *J* = 8 Hz, 1H, Ar-H), 7.56 (t, 1H, Ar-H), 7.53 (d, *J* = 3.2 Hz, 2H, Ar-H), 7.49 (d, *J* = 8 Hz, 2H, Ar-H), 7.43 (t, 1H, Ar-H), 7.34 (d, *J* = 8 Hz, 2H, Ar-H), 2.70 (s, 3H, CH_3_); ^13^C-NMR (DMSO-*d*_6_) δ: 154.00, 149.00, 147.50, 139.00, 138.80, 137.00, 135.00, 129.50, 129.00, 128.00, 127.50, 127.00, 126.00, 125.00, 123.00, 120.00, 117.70, 22.00; FT-IR (ύ max, cm^−1^): 3464 (3 NH), 3059 (CH aromatic), 1635 (C=O amide); MS (MWt.: 403.86): *m*/*z*, 405.04 [M^+^ + 2,(1.28%) ], 403.89 [M^+^, (0.9%)], 89.99 (100%); Anal. calcd for C_22_H_18_ClN_5_O: C, 65.43; H, 4.49; N, 17.3. Found: C, 65.58; H, 4.57; N, 17.51.

#### 3.2.23. 1-(4-((3-Methylquinoxalin-2-yl)amino)phenyl)-3-(m-tolyl)urea (**VIIId**) 

Black solid (yield = 65%); m.p. 280–281 °C; ^1^H-NMR (DMSO-*d*_6_) δ: 9.18 (s, 1H, NH urea), 8.57 (s, 1H, NH urea), 8.56 (s, 1H, NH), 7.85 (d, *J* = 8 Hz, 1H, Ar-H), 7.71–7.46 (m, 4H, Ar-H), 7.35 (s, 1H, Ar-H), 7.31 (m, 1H, Ar-H), 7.23 (d, *J* = 8 Hz, 2H, Ar-H), 7.14 (t, 1H, Ar-H), 6.78 (d, *J* = 8 Hz, 2H, Ar-H), 2.49 (s, 3H, CH_3_), 2.27 (s, 3H, CH_3_); FT-IR (ύ max, cm^−1^): 3456 (3 NH), 3035 (CH aromatic), 2958 (CH aliphatic), 1639 (C=O amide); MS (MWt: 383.45): *m*/*z*, 384.12 [M^+^ + H, (3.19%)], 383.12 [M^+^, (12.44%)], 249.08 (100%); Anal. calcd for C_23_H_21_N_5_O: C, 72.04; H, 5.52; N, 18.26. Found: C, 72.23; H, 5.61; N, 18.42.

#### 3.2.24. 1-(3-Methoxyphenyl)-3-(4-((3-methylquinoxalin-2-yl)amino)phenyl)urea (**VIIIe**) 

Black solid (yield = 79%); m.p. 228–230 °C; ^1^H-NMR (DMSO-*d*_6_) δ: 8.68 (s, 1H, NH urea), 8.60 (s, 1H, NH urea), 8.57 (s, 1H, NH), 7.86 (d, *J* = 8 Hz, 2H, Ar-H), 7.79 (d, *J* = 8 Hz, 1H, Ar-H), 7.64 (d, *J* = 8 Hz, 1H, Ar-H),7.53 (t, 1H, Ar-H), 7.47 (d, *J* = 8 Hz, 2H, Ar-H), 7.42 (t, 1H, Ar-H), 7.38 (s, 1H, Ar-H), 7.22–7.20 (m, 1H, Ar-H), 7.18 (d, *J* = 8 Hz, 1H, Ar-H), 6.95 (d, *J* = 8 Hz, 1H, Ar-H), 3.74 (s, 3H, OCH_3_), 2.71 (s, 3H, CH_3_); ^13^C-NMR (DMSO-*d*_6_) δ: 160.00, 153.00, 149.00, 146.00, 142.00, 141.00, 137.00, 136.00, 135.00, 130.00, 129.00, 127.00, 126.00, 125.00, 123.00, 118.00, 111.00, 109.00, 105.00, 55.00, 22.00; FT-IR (ύ max, cm^−1^): 3298 (3 NH), 3059 (CH aromatic), 2935 (CH aliphatic),1639 (C=O amide); MS (MWt: 399.45): *m*/*z*, 400.85 [M^+^ + H, (2.88%) ], 399.24 [M^+^, (11.49%) ], 54.07 (100%); Anal. calcd for C_23_H_21_N_5_O_2_: C, 69.16; H, 5.30; N, 17.53. Found: C, 69.41; H, 5.37; N, 17.65.

#### 3.2.25. 1-(4-((3-Chloroquinoxalin-2-yl)amino)phenyl)-3-phenylthiourea (**XVIIa**) 

Brown solid (yield = 62%); m.p. 190–191 °C; ^1^H-NMR (DMSO-*d*_6_) δ: 11.00 (s, 1H, NH thiourea), 10.60 (s, 1H, NH thiourea), 9.90 (s, 1H, NH), 8.04–7.90 (m, 3H, Ar-H), 7.60–7.50 (m, 4H, Ar-H), 7.40–7.30 (m, 5H, Ar-H), 7.29–7.15 (m, 1H, Ar-H); FT-IR (ύ max, cm^−1^): 3371 (3 NH), 3059 (CH aromatic), 1238 (C=S amide); MS (MWt: 405.9): *m*/*z*, 407.11 [M^+^ + 2, (3.9%)], 405.07 [M^+^, (4.2%)], 45.04 (100%); Anal. Calcd for C_21_H_16_ClN_5_S: C, 62.14; H, 3.97; N, 17.25. Found: C, 62.37; H, 4.03; N, 17.47.

#### 3.2.26. 1-(4-((3-Chloroquinoxalin-2-yl)amino)phenyl)-3-phenylurea (**XVIIb**) 

Brown solid (yield = 58%); m.p. 280–281 °C; ^1^H-NMR (DMSO-*d*_6_) δ: 11.00 (s, 1H, NH urea), 10.60 (s, 1H, NH urea), 9.90 (s, 1H, NH), 8.10 (m, 1H, Ar-H), 8.03–7.94 (m, 3H, Ar-H), 7.60–7.50 (m, 4H, Ar-H), 7.42–7.30 (m, 4H, Ar-H), 7.15 (m, 1H, Ar-H). FT-IR (ύ max, cm^−1^): 3302 (3 NH), 3059 (CH aromatic), 1635 (C=O amide); MS (MWt: 389.84): *m*/*z*, 391.25 [M^+^ + 2, (4.94%)], 389.46 [M^+^, (6.21%)], 55.19 (100%); Anal. Calcd for: C_21_H_16_ClN_5_O: C, 64.70; H, 4.14; N, 17.96. Found: C, 64.86; H, 4.17; N, 18.13.

#### 3.2.27. 1-(4-Chlorophenyl)-3-(4-((3-chloroquinoxalin-2-yl)amino)phenyl)urea (**XVIIc**)

Olive green solid (yield = 63%); m.p.> 300 °C; ^1^H-NMR (DMSO-*d*_6_) δ: 9.70 (s, 1H, NH urea), 9.50 (s, 1H, NH urea), 9.01 (s, 1H, NH), 8.00–7.90 (m, 1H, Ar-H), 7.59–7.47 (m, 5H, Ar-H), 7.39–7.31 (m, 6H, Ar-H); FT-IR (ύ max, cm^−1^): 3294 (3 NH), 3066 (CH aromatic), 1635 (C=O amide); MS (MWt: 424.28): *m*/*z*, 428.32 [M^+^ + 4, (0.7%)], 424.14 [M^+^, (2.59%)], 60.07 (100%); Anal. Calcd for C_21_H_15_C_l2_N_5_O: C, 59.45; H, 3.56; N, 16.51. Found: C, 59.68; H, 3.54; N, 16.79.

#### 3.2.28. 1-(4-((3-Chloroquinoxalin-2-yl)amino)phenyl)-3-(m-tolyl) urea (**XVIId**)

Dark brown solid (yield = 77%); m.p. > 300 °C; ^1^H-NMR (DMSO-*d*_6_) δ: 9.05 (s, 1H, NH urea), 8.63 (s, 1H, NH urea), 8.58 (s, 1H, NH), 7.96 (m, 2H, Ar-H), 7.84–7.79 (m, 2H, Ar-H), 7.58–7.53 (m, 1H, Ar-H), 7.51 (d, *J* = 8 Hz, 2H, Ar-H), 7.32 (s, 1H, Ar-H), 7.27 (d, *J* = 8 Hz, 2H, Ar-H), 7.17 (t, 1H, Ar-H), 6.80 (d, *J* = 8 Hz, 1H, Ar-H), 2.29 (s, 3H, CH_3_); FT-IR (ύ max, cm^−1^): 3298 and 3194 (3 NH), 2954 (CH aliphatic), 1635 (C=O amide); MS (MWt: 403.86): *m*/*z*, 405.33 [M^+^ + 2, (1.15%)], 403.2 [M^+^, (3.24%)], 63 (100%); Anal. Calcd for C_22_H_18_ClN_5_O: C, 65.43; H, 4.49; N, 17.34. Found: C, 65.64; H, 4.58; N, 17.46.

#### 3.2.29. 1-(4-((3-Chloroquinoxalin-2-yl)amino)phenyl)-3-(3-methoxyphenyl) urea (**XVIIe**)

Olive green solid (yield = 76%); m.p. >300 °C; ^1^H-NMR (DMSO-*d*_6_) δ: 9.57 (s, 1H, NH urea), 9.16 (s, 1H, NH urea), 8.74 (s, 1H, NH), 8.01–7.90 (m, 3H, Ar-H), 7.80 (d, *J* = 8 Hz, 1H, Ar-H), 7.52–7.42 (m, 3H, Ar-H), 7.27–7.25 (m, 3H, Ar-H), 7.16–7.08 (m, 2H, Ar-H), 3.68 (s, 3H, OCH_3_); FT-IR (ύ max, cm^−1^): 3310 (3 NH), 3030 (CH aromatic), 2920 (CH aliphatic), 1639 (C=O amide), 1215 (OCH_3_); MS (MWt: 419.86): *m*/*z*, 419.27 [M^+^, (2.52%)], 83.98 (100%); Anal. Calcd for C_22_H_18_ClN_5_O_2_: C, 62.93; H, 4.32; N, 16.68. Found: C, 63.14; H, 4.36; N, 16.89.

#### 3.2.30. 1-(3-Methoxyphenyl)-3-(4-(quinoxalin-2-ylamino)phenyl)urea (**XXV**)

Brown solid (yield = 61%); m.p. > 300 °C; ^1^H-NMR (DMSO-*d*_6_): δ: 9.67 (s, 1H, NH urea), 9.57 (s, 1H, NH urea), 8.64 (s, 1H, NH), 7.38–7.12 (m, 5H, Ar-H), 7.02–6.91 (m, 4H, Ar-H), 6.67–6.54 (m, 4H, Ar-H), 3.79 (s, 3H, OCH_3_). FT-IR (ύ max, cm^−1^): 3286 (3 NH), 3066 (CH aromatic), 2924 (CH aliphatic), 1716 (C=O amide); MS (MWt: 385.42): *m*/*z*, 385.4[M^+^, (1.6%)], 118.98 (100%); Anal. Calcd for C_22_H_19_N_5_O_2_: C, 68.56; H, 4.97; N, 18.17. Found: C, 68.79; H, 5.12; N, 18.43.

#### 3.2.31. 1-(4-((3-Chloroquinoxalin-2-yl)amino)phenyl)-3-(4-methoxyphenyl)urea (**XVIIf**)

*p*-Methoxy- benzoyl azide (10 mmol) was heated in dry toluene (100 mL) at 60 °C for half an hour, then intermediate **XIII** (10 mmol) in dry toluene (5 mL) was added dropwise to the solution and the reaction was refluxed for additional 5 h [32]. The solid formed was filtered, washed with toluene and dried to give the urea derivative **XVIIf**. Light green solid (yield = 71%); m.p. > 300 °C; ^1^H-NMR (DMSO-*d*_6_) δ: 10.12 (s, 1H, NH urea), 9.58 (s, 1H, NH urea), 8.80 (s, 1H, NH), 8.58 (d, *J* = 12 Hz, 1H, Ar-H), 8.05 (d, *J* = 16 Hz, 1H, Ar-H), 7.88 (m, 1H, Ar-H), 7.51 (m, 2H, Ar-H), 7.37 (m, 4H, Ar-H), 6.89 (m, 3H, Ar-H), 3.86 (s, 3H, OCH_3_); FT-IR (ύ max, cm^−1^): 3309 (3 NH), 3066 (CH aromatic), 2927 (CH aliphatic), 1639 (C=O amide); MS (MWt: 419.86): *m*/*z*, 420 [M^+^ + H, (0.7%)], 419.42 [M^+^, (0.96%)], 139 (100%); Anal. Calcd for C_22_H_18_ClN_5_O_2_: C, 62.93; H, 4.32; N, 16.68. Found: C, 63.12; H, 4.37; N, 16.85.

#### 3.2.32. General Procedures for the Synthesis of Compounds **IXa**,**b** and **XVIIIa**,**b**

To a solution of **IV** or **XIII** (0.35 mmol) in dry pyridine (10 mL) the appropriate sulfonyl chloride (1.05 mmol; 3 eq.) namely (4-methylbenzenesulfonyl chloride, 4-nitrobenzenesulfonyl chloride) was added and the mixture was refluxed for 2 h, poured onto cold water (30 mL) and stirred for 30 min [33]. The resulted solid was filtered, washed with water, dried and recrystallized from ethanol to afford the title compounds **IXa**,**b** or **XVIIIa**,**b**, respectively.

#### 3.2.33. 4-Methyl-*N*-(4-((3-methylquinoxalin-2-yl)amino)phenyl) benzenesulphonamide (**IXa**)

Dark green solid (yield = 75%); m.p. 198–199 °C; ^1^H-NMR (DMSO-*d*_6_) δ: 10.70 (s, 1H, NH sulfonamide), 8.63 (s, 1H, NH), 8.54–6.70 (m, 12H, Ar-H), 2.72 (s, 3H, CH_3_), 2.32 (s, 3H, CH_3_); FT-IR (ύ max, cm^−1^): 3421 (2 NH), 1580 (NH bending), 1335 and 1160 (SO_2_); MS (MWt.: 404.5): *m*/*z*, 405 [M^+^ + H, (2.74%)], 404.08 [M^+^, (9.05%)], 249 (100%); Anal. calcd for C_22_H_20_N_4_O_2_S: C, 65.33; H, 4.98; N, 13.85. Found: C, 65.49; H, 5.06; N, 13.97.

#### 3.2.34. *N*-(4-((3-Methylquinoxalin-2-yl)amino)phenyl)-4-nitrobenzenesulfonamide (**IXb**)

Black solid (yield = 70%); m.p. 180–182 °C; ^1^H-NMR (DMSO-*d*_6_) δ: 8.63 (s,1H, NH sulfonamide), 8.32 (s, 1H, NH), 8.22 (d, *J* = 8 Hz, 2H,Ar-H), 7.95 (d, *J* = 4 Hz, 2H, Ar-H), 7.85 (d, *J* = 8 Hz, 2H, Ar-H), 7.78 (d, *J* = 8 Hz, 2H, Ar-H), 7.71 (d, *J* = 8 Hz, 1H, Ar-H), 7.58 (m, 1H, Ar-H), 7.44 (t, 1H, Ar-H), 7.36 (t, 1H, Ar-H), 2.73 (s, 3H, CH_3_); FT-IR (ύ max, cm^−1^): 3414 (2 NH), 3035 (CH aromatic), 2924 (CH aliphatic), 1604 (NH bending), 1346 and 1165 (SO_2_); MS (MWt: 435.46): *m*/*z*, 435.42 [M^+^, (10.24%)], 44.88 (100%); Anal. calcd for C_21_H_17_N_5_O_4_S: C, 57.92; H, 3.93; N, 16.08. Found: C, 58.09; H, 3.97; N, 16.23.

#### 3.2.35. *N*-(4-((3-Chloroquinoxalin-2-yl)amino)phenyl)-4-methylbenzenesulfonamide (**XVIIIa**)

Light green solid (yield = 75%); m.p. 250–252 °C; ^1^H-NMR (DMSO-*d*_6_) δ: 10.21 (s, 1H, NH sulfonamide), 9.89 (s, 1H, NH), 8.02–7.91 (m, 4H, Ar-H), 7.85 (d, *J* = 8 Hz, 2H, Ar-H), 7.64–7.57 (m, 2H, Ar-H), 7.36 (d, *J* = 8 Hz, 4H, Ar-H), 2.37 (s, 3H, CH_3_); FT-IR (ύ max, cm^−1^): 3386 (2 NH), 3083 (CH aromatic), 2921 (CH aliphatic), 1332–1159 (SO_2_); MS (MWt: 424.9): *m*/*z*, 425 [M^+^ + H, (1%)], 424.7 [M^+^, (1.58%)], 261 (100%); Anal. Calcd for C_21_H_17_ClN_4_O_2_S: C, 59.36; H, 4.03; N, 13.19. Found: C, 59.48; H, 4.12; N, 13.34.

#### 3.2.36. *N*-(4-((3-Chloroquinoxalin-2-yl)amino)phenyl)-4-nitrobenzenesulfonamide (**XVIIIb**)

Dark brown solid (yield = 76%); m.p. > 300 °C, ^1^H-NMR (DMSO-*d*_6_) δ: 10.46 (s, 1H, NH sulfonamide), 9.40 (s, 1H, NH), 8.42–8.19 (m, 2H, Ar-H), 8.00–7.83 (m, 5H, Ar-H), 7.60-7.46 (m, 2H, Ar-H), 7.35–7.13 (m, 3H, Ar-H); FT-IR (ύ max, cm^−1^): 3385 (3 NH), 3053 (CH aromatic), 1347–1165 (SO_2_); MS (MWt.: 455.9): *m*/*z*, 455.68 [M^+^, (0.77%)], 107 (100%); Anal. Calcd for C_20_H_14_ClN_5_O_4_S: C, 52.69; H, 3.10; N, 15.36. Found: C, 52.85; H, 3.07; N, 15.48.

### 3.3. Biological Studies

#### 3.3.1. In-Vitro Anti-Cancer Activity

Three cancer cell lines including human colon carcinoma (HCT116), human breast adenocarcinoma (MCF-7) and human hepatocellular carcinoma (Hep G2), were purchased from American Type Cell Culture Collection (ATCC, Manassas, VA, USA) and grown on the appropriate growth medium Dulbecco’s modified Eagle’s medium (DMEM) or Roswell Park Memorial Institute medium (RPMI 1640) supplemented with 100 mg/mL of streptomycin, 100 units/mL of penicillin and 10% of heat-inactivated fetal bovine serum in a humidified, 5% (*v*/*v*) CO_2_ atmosphere at 37 °C [34]. Cytotoxicity assay by 3-[4,5-dimethylthiazole-2-yl]-2,5-diphenyltetrazolium bromide (MTT).

Exponentially growing cells from different cancer cell lines were trypsinized, counted and seeded at the appropriate densities (2000–1000 cells/0.33 cm^2^ well) into 96-well microtiter plates. Cells then were incubated in a humidified atmosphere at 37 °C for 24 h. Then, cells were exposed to different concentrations of compounds (0.1, 10, 100, 1000 µM) for 72 h. Then the viability of treated cells was determined using MTT technique as follow. Media were removed; cells were incubated with 200 μL of 5% MTT solution/well (Sigma Aldrich, Darmstadt, Germany) and were allowed to metabolize the dye into a colored-insoluble formazan crystal for 2 h. The remaining MTT solution were discarded from the wells and the formazan crystals were dissolved in 200 µl/well acidified isopropanol for 30 min, covered with aluminum foil and with continuous shaking using a MaxQ 2000 plate shaker (Thermo Fisher Scientific Inc, Waltham, MA, USA) at room temperature. Absorbance were measured at 570 nm using a Stat Fax^R^ 4200 plate reader (Awareness Technology, Inc., Awareness Technology, FL, USA). The cell viability was expressed as percentage of control and the concentration that induces 50% of maximum inhibition of cell proliferation (IC_50_) were determined using Graph Pad Prism version 5 software (Graph Pad Software Inc, San Diego, CA, USA) (1,2) [35].

#### 3.3.2. In-Vitro VEGFR-2 Inhibition Assay

The in-vitro enzyme inhibition determination for the synthesized compounds was carried out at Bio Science Corporation (BPS)-San Diego, CA, USA. The assay was performed using Kinase-Glo Plus luminescence kinase assay kit (Promega), where the kinase activity was measured by quantitating the amount of ATP remaining in solution following a kinase reaction. The luminescent signal from the assay is correlated with the amount of ATP present and is inversely correlated with the amount of kinase activity. Our compounds were diluted with 10% DMSO and 5 µL of the dilution was added to a 50 µL reaction so that the final concentration of DMSO is 1% in all of reactions. All of the enzymatic reactions were conducted at 30 °C for 40 min. The 50 µL reaction mixture contains 40 mM Tris, pH 7.4, 10 mM MgCl_2_, 0.1 mg/mL BSA, 1 mM DTT, 10 µM ATP, Kinase substrate and the enzyme. After the enzymatic reaction, 50 µL of Kinase-Glo Plus Luminescence kinase assay solution (Promega San Diego, CA, USA) was added to each reaction and incubate the plate for 5 min at room temperature. Luminescence signal was measured using a BioTek Synergy 2 microplate reader.

Kinase activity assays were performed in duplicate at each concentration. The luminescence data were analyzed using the computer software, Graphpad Prism. The difference between luminescence intensities in the absence of Kinase (Lu_t_) and in the presence of Kinase (Lu_c_) was defined as 100% activity (Lu_t_ − Lu_c_). Using luminescence signal (Lu) in the presence of the compound, % activity was calculated as:

% activity = {(Lu_t_ − Lu)(Lu_t_ − Lu_c_)} × 100%

where Lu = the luminescence intensity in the presence of the compound.

#### 3.3.3. Cell Cycle Analysis

HCT116 cells at a density of 4 × 10^6^ cell/T 75 flask were exposed to 1-(4-Chlorophenyl)-3-(4-((3-methylquinoxalin-2-yl)amino)phenyl)urea **VIIIc** at its IC_50_ concentration for 24 and 48 h. The cells then were collected by trypsinization, washed with phosphate buffered saline (PBS), and fixed in ice-cold absolute alcohol. Thereafter, cells were stained, using Cycle test ^TM^ Plus DNA Reagent Kit (BD Biosciences, San Jose, CA, USA), according to the manufacturer’s instructions. Cell cycle distribution was determined using a FACS Calibur flow cytometer (BD Biosciences).

#### 3.3.4. Apoptosis Determination

Apoptosis was determined by staining cells with Annexin V–fluorescein isothiocyanate (FITC) and counterstaining with propidium iodide (PI) using the Annexin V–FITC/PI apoptosis detection kit (BD PharMingen, San Diego, CA, USA) according to the manufacturer’s instructions. Briefly, 4 × 10^6^ cell/T 75 flask were exposed to compound **VIIIc** at its IC_50_ concentration for 24 and 48 h. The cells then were collected by trypsinization and 0.5 × 10^6^ cells were washed twice with phosphate-buffered saline (PBS) and stained with 5 μL Annexin V–FITC and 5 μL PI in 1× binding buffer (BD PharMingen) for 15 min at room temperature in the dark. Analyses were performed using FACS Calibur flow cytometer (BD Biosciences).

#### 3.3.5. In Vitro Cytotoxic Assay

Cell Line cells were obtained from American Type Culture Collection, cells were cultured using DMEM (Invitrogen/Life Technologies, Waltham, MA, USA) supplemented with 10% FBS (Hyclone, Waltham, MA, USA), 10 μg/mL of insulin (Sigma), and 1% penicillin-streptomycin. All of the other chemicals and reagents were from Sigma, or Invitrogen. Plate cells (cells density 1.2–1.8 × 10,000 cells/well) in a volume of 100 µL complete growth medium + 100 μL of the tested compound per well in a 96-well plate for 24 h before the MTT assay.

## 4. Conclusions

The present paper aimed to design some quinoxaline scaffolds and screened them for their cytotoxicity against three tumor cell lines and as well as measuring their selectivity. Compound **VIIIc** showed promising activity against human colon carcinoma (HCT116) cell line (IC_50_ = 2.5 µM), also it induced a significant disruption in cell cycle profile and cell cycle arrest at G2/M phase boundary with concurrent time dependent increase in pre-G cell population. Owing to the good anti-cancer activity of compound **VIIIc** toward (HCT116) cells and its selectivity, it could be considered as a lead compound that merits further optimization and development as an anti-cancer and an apoptotic inducing candidate against (HCT116) cells.

## Figures and Tables

**Table 1:**
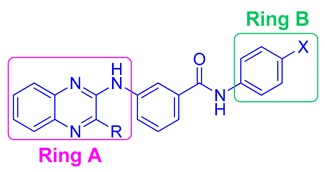
Results of in-vitro anti-cancer activity of *N*-(phenyl)-3-(quinoxalin-2-ylamino) benzamide derivatives screened against three tumor cell lines.

Compound	Ring A (R)	Ring B (X)	HCT116 Cell Line IC_50_ (µM)	Hep G2 Cell Line IC_50_ (µM)	MCF-7 Cell Line IC_50_ (µM)
**VIa**	CH_3_	H	602.5	>1000	692
**VIb**	CH_3_	Cl	537	955	692
**VIc**	CH_3_	OCH_3_	275	479	288
**XVa**	**Cl**	**H**	**4.4**	**10**	**5.3**
**XVb**	Cl	Cl	15.5	27.5	21.4
**XVc**	Cl	OCH_3_	182	912	380
**XXIVa**	H	Cl	219	436.5	182
**XXIVb**	H	OCH_3_	302	490	219
**Doxorubicin**			0.62	1.2	0.9

**Table 2:**
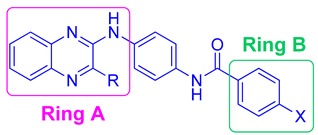
Results of in-vitro anti-cancer activity of *N*-(4-(quinoxalin-2-yl)amino)phenyl)benzamide derivatives screened against three tumor cell lines.

Compound	Ring A (R)	Ring B (X)	HCT116 Cell Line IC_50_ (µM)	Hep G2 Cell Line IC_50_ (µM)	MCF-7 Cell Line IC_50_ (µM)
**VIIa**	CH_3_	H	21.9	27.5	25.7
**VIIb**	CH_3_	Cl	27.5	114.8	138
**VIIc**	CH_3_	OCH_3_	33.9	19	24
**VIId**	**CH_3_**	**CH_3_**	**7.8**	**25.7**	**60.3**
**XVIa**	Cl	H	346.7	512.9	323.6
**XVIb**	Cl	Cl	>1000	>1000	>1000
**XVIc**	Cl	4-OCH_3_	>1000	>1000	>1000
**Doxorubicin**			0.62	1.2	0.9

**Table 3:**
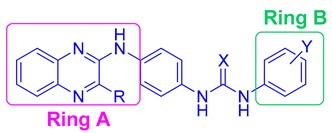
Results of in-vitro anti-cancer activity of 1-phenyl-3-(4-(quinoxalin-2-yl)amino)phenyl) thiourea and urea derivatives screened against three tumor cell lines.

Compound	Ring A R	X	Ring B Y	HCT116 Cell Line IC_50_ (µM)	Hep G2 Cell Line IC_50_ (µM)	MCF-7 Cell Line IC_50_ (µM)
**VIIIa**	CH_3_	S	H	12.3	9.8	15.5
**VIIIb**	CH_3_	O	H	38	98	13.5
**VIIIc**	**CH_3_**	**O**	**4-Cl**	**2.5**	**22**	**9**
**VIIId**	CH_3_	O	3-CH_3_	11	36	30
**VIIIe**	**CH_3_**	**O**	**3-OCH_3_**	**8.4**	**21.4**	**24.5**
**XVIIa**	Cl	S	H	309	575	170
**XVIIb**	Cl	O	H	170	513	148
**XVIIc**	Cl	O	4-Cl	257	616.5	692
**XVIId**	Cl	O	3-CH_3_	190.5	513	64.5
**XVIIe**	Cl	O	3-OCH_3_	40.7	55	47.9
**XVIIf**	Cl	O	4-OCH_3_	457	724	436.5
**XXV**	H	O	3-OCH_3_	31	64.5	32
**Doxorubicin**				0.62	1.2	0.9

**Table 4:**
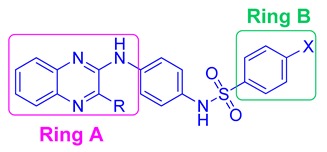
Results of in-vitro anti-cancer activity of *N*-(4-(quinoxalin-2-yl)amino)phenyl)substituted benzene sulfonamide derivatives screened against three tumor cell lines.

Compound	R	X	HCT116 Cell Line IC_50_ (µM)	Hep G2 Cell Line IC_50_ (µM)	MCF-7 Cell Line IC_50_ (µM)
**IXa**	CH_3_	CH_3_	21.9	22.9	22.9
**IXb**	CH_3_	NO_2_	12	24.5	10.23
**XVIIIa**	Cl	CH_3_	302	489.8	537
**XVIIIb**	Cl	NO_2_	>1000	>1000	55
**Doxorubicin**			0.62	1.2	0.9

**Table 5:** Percentage of inhibition of VEGFR-2 with 10 µM of the synthesized compounds.

Compounds	% of VEGFR-2 Inhibition	Compounds	% of VEGFR-2 Inhibition	Compounds	% of VEGFR-2 Inhibition
**VIIa**	2	**VIIIa**	7	**VIa**	7
**XVIa**	8	**XVIIa**	10	**XVb**	7
**VIIb**	4	**VIIIb**	4	**XVc**	5
**XVIb**	4	**XVIIb**	10	**VIc**	5
**VIIc**	6	**XVIIf**	6	**XXIVb**	9
**XVIc**	4	**VIIIc**	7	**XXIVa**	3
**VIId**	3	**XVIIc**	6	**XXV**	21
**XVId**	3	**VIIId**	5	**Staurosporine**	100
**IXa**	3	**XVIId**	5		
**XVIIIa**	4	**VIIIe**	6		
**IXb**	3	**XVIIe**	8		
**XVIIIb**	9	**XVa**	6		

**Table 6:** Values of IC_50_ for the most active compounds on WI-38 cell line.

Compound	IC_50_ (µM)
**XVa**	**163.6 ± 9.2**
**VIIIa**	**126.19 ± 6.81**
**VIIIc**	97.2 ± 5.66
**VIId**	40.13 ± 3.42
**VIIIe**	96.4 ± 5.72
**staurosporine**	101.86 ± 6.34
